# Family functionality and social support in Brazilian older people with chronic kidney disease in hemodialysis: a cross-sectional study

**DOI:** 10.1590/1516-3180.2025.3529.18032026

**Published:** 2026-07-10

**Authors:** Ana Beatriz Murari Thomazini, Diana Gabriela Mendes dos Santos, Yngrid Larisse Teodoro de Souza, Daiane Baldo Apolinário, Ana Luiza Barbosa, Marisa Silvana Zazzetta, Fabiana de Souza Orlandi

**Affiliations:** I Department of Gerontology, Universidade Federal de São Carlos (UFSCAR), São Carlos (SP), Brazil.; II Department of Nursing, Universidade Federal de São Carlos (UFSCAR), São Carlos (SP), Brazil; III Department of Gerontology, Universidade Federal de São Carlos (UFSCAR), São Carlos (SP), Brazil.; IV Department of Gerontology, Universidade Federal de São Carlos (UFSCAR), São Carlos (SP), Brazil.; V Department of Gerontology, Universidade Federal de São Carlos (UFSCAR), São Carlos (SP), Brazil.; VI Department of Gerontology, Universidade Federal de São Carlos (UFSCAR), São Carlos (SP), Brazil.; VII Department of Gerontology, Universidade Federal de São Carlos (UFSCAR), São Carlos (SP), Brazil.

**Keywords:** Renal insufficiency, chronic, Renal dialysis, Social support, Family support, Chronic kidney disease, Hemodialysis, Social support

## Abstract

**BACKGROUND:**

End-stage chronic kidney disease (CKD) requires hemodialysis, which carries significant biopsychosocial impact.

**OBJECTIVES:**

This study aimed to investigate the relationship between family functionality and social support among older individuals undergoing hemodialysis.

**DESIGN AND SETTING:**

This cross-sectional and correlational study was conducted at a renal replacement therapy unit located in São Paulo State.

**METHODS:**

A total of 118 participants aged ≥ 60 years undergoing hemodialysis were interviewed individually using the Family adaptability, partnership, growth, affection, and resolve (APGAR), and the Medical Outcomes Study Social Support Scale. All ethical principles were duly respected.

**RESULTS:**

Majority of participants were male (69.5%), aged between 60–69 years (50.8%), white ethnicity (63.6%), with a stable partner (54.2%), and had an income of one to three times the minimum wage (55.9%). Regarding the domains of social support, affective support (AS) had the highest mean score (93.44 ± 12.97), whereas informational support (IS) had the lowest (81.69 ± 24.60). Overall, 83.9% reported good family functioning, 9.3% moderate family dysfunction, and 6.8% severe dysfunction. A statistically significant positive correlation with a moderate magnitude was confirmed between APGAR with all support domains. Material support (MS, *r* = 0.327), AS (*r* = 0.425), emotional support (ES, *r* = 0.518), positive social interaction support (PSIS, *r* = 0.466), and IS (*r* = 0.421).

**CONCLUSION:**

Social support was positively associated with family functioning, indicating that patients with CKD undergoing hemodialysis who reported better family functioning also perceived higher levels of social support.

## INTRODUCTION

Noncommunicable diseases (NCDs) are long-lasting conditions arising from a mix of inherited traits, physiological functions, lifestyle choices, and environmental influences. According to the World Health Organization (WHO),^1^ NCDs account for a disproportionate burden in low- and middle-income countries, where approximately 75% of the global mortality is attributed to these conditions. NCDs include chronic kidney disease (CKD), which affects approximately 10% of the global population. According to WHO estimates, CKD affects an estimated 6.7% of adults in Brazil, with prevalence approximately tripling among older adults aged 60 years and older.^2^

According to the kidney disease: Improving Global Outcomes CKD Work Group,^3^ CKD is characterized by structural or functional abnormalities of the kidneys that persist for a minimum of three months with health implications on the individual. The classification of CKD relies on three key parameters to establish a definitive diagnosis: the cause, glomerular filtration rate, and albuminuria level. Patients with end-stage CKD require treatment, and hemodialysis is one of the main treatment modalities. Although hemodialysis improves health status and prolongs patient survival, it neither halts disease progression nor fully reproduce the normal kidney function.^4,5^

Individuals receiving hemodialysis treatment must follow strict therapeutic routines that demand significant changes to their daily habits and lifestyle, with important emotional, social, and family repercussions. Given this context, social support is a key determinant to enhance the quality of life among individuals receiving hemodialysis. Sułkowski et al.^4^ highlighted social support as an essential element in reducing the negative impact of CKD in patients undergoing hemodialysis and in promoting better health outcomes, especially among older individuals.^6^

Considering the above, family support is also crucial during hemodialysis as it can influence both health status and quality of life. According to Hulu et al.,^7^ greater family support leads to better the quality of life of the patient. Close relationships with family, friends, and other significant individuals play a central role in shaping the support patients received, and higher family resilience are associated with increased support. In addition, evidence suggests that among older individuals with CKD undergoing hemodialysis, stronger and more consistent family involvement is associated with improved psychological outcomes.^5,8^

Despite the recognized importance of social and familial support for older adults undergoing hemodialysis, research from Brazil in this area remains limited. Few studies, such as those by Santos et al.^9^ and Wang et al.^10^ have simultaneously examined family functioning and social support in this population, and even fewer have employed adjusted analyses or longitudinal designs. This gap highlights the need for a deeper investigation into how these psychosocial factors interact and influence patient outcomes over time.

## OBJECTIVE

This study aimed to investigate the relationship between family functionality and social support among older individuals undergoing hemodialysis in the Brazilian context.

## METHODS

### Study design and population

This cross-sectional and correlational study with a quantitative approach was conducted at a renal care facility in the interior of the state of São Paulo, Brazil.

Clinical data were collected from February to August 2025. Participants were recruited through non-probabilistic consecutive sampling based on the inclusion criteria of being 60 years or older, undergoing hemodialysis treatment for at least 6 months and whose oral communication ability was preserved. The exclusion criteria was a documented diagnosis of dementia in the medical chart that would make the interview unfeasible. For the sample size calculation, a correlation coefficient value of *r* ≥ 0.40 was considered, given that *r* = 0.40 is indicative of a moderate correlation between psychosocial variables according to Ajzen & Fishbein,^11^ and with the fixed values of α = 0.05 and β = 0.05. This estimation yielded a minimum required number of 75 participants.

### Data collection and definitions

At the beginning of participants’ interactions, the study was explained to eligible participants, who were then invited to take part; those who agreed were provided with informed consent form. Subsequently, an individual interview was conducted using a structured instrument that included a sociodemographic, socioeconomic, and health conditions questionnaire, the medical outcomes study (MOS) social support assessment instrument; and the Family adaptability, partnership, growth, affection, and resolve (APGAR) to assess family functioning.

The dependent variable was social support, assessed using the MOS Social Support Scale developed by Sherbourne and Stewart^12^ and validated and adapted for use in Brazil by Andrade.^13^ The instrument consists of 19 items divided into five categories of support: tangible, affectionate, emotional, positive social interaction, and informational support. Each support type includes four questions, with five response options: 1 = “Never,” 2 = “Rarely,” 3 = “Sometimes,” 4 = “Very frequently,” and 5 = “Always.” Each response contributes to the individual’s perceived level of support. For four of the support domains, categories are summed and divided by 20, and the resulting value is multiplied by 100. Affectionate support is scored differently, as it includes only three questions; the total score is divided by 15 and then multiplied by 100. The final scores for each support category range from 20 to 100, with higher scores indicating greater perceived support.

The principal independent variable was family functionality, evaluated using the Family APGAR scale developed by Smilkstein^14^ and validated in Brazil by Duarte.^15^ The instrument consists of five questions, each offering five response options: 0 = “Never,” 1 = “Rarely,” 2 = “Sometimes,” 3 = “Very Frequently,” and 4 = “Always.” The final score, ranging from 1 to 20 (where scores ≥ 12 indicate good functioning and scores < 12 suggest dysfunction), was operationalized as the independent variable in two formats for statistical analysis: the total raw score was treated as a continuous variable and utilized for comparisons of means and medians of social support, whereas the dichotomized score (functional vs. dysfunctional) was used as categorical variable and was employed to calculate Spearman’s correlation with social support.

The sociodemographic and socioeconomic questionnaire included items related to age, sex, ethnicity, education level, income, and marital status. 

### Statistical analysis

The statistical analysis of the data was performed using SPSS software, v.22.0 (IBM Inc., Chicago, Illinois, United States). Descriptive analyses were performed, including central trend data (mean, minimum, and maximum) and dispersion measures (standard deviation). The Kolmogorov–Smirnov test was used to verify the absence of data normality and nonparametric tests were adopted.

To compare the social support scores, according to the levels of family functioning, the Kruskal–Wallis test was adopted. Spearman’s correlation coefficients were calculated to verify the relationship between the continuous variables. In the present study, the magnitude of correlations was classified according to Levin and Fox’s proposition (2004): weak (< 0.3); moderate (0.3 to 0.59); (0.6 to 0.9), perfect (1.0). The significance level adopted for the statistical tests was 5% (*P* ≤ 0.05).^16^

### Ethical Considerations

All ethical principles were respected, and the research project was approved prior to the start of the study by the Human Research Ethics Committee of the Universidade Federal de São Carlos (UFSCAR; approval no. 7.130.768; CAAE: 80181224.5.0000.5504).

## RESULTS

This study included 118 older patients undergoing hemodialysis treatment who met the predefined eligibility criteria. Regarding participants’ characterization, 82 were men (69.5%), 60 were aged between 60– 69 years (50.8%), 75 were white (63.6%), and 64 reported having a stable partner (54.2%). Regarding income, 66 individuals reported a monthly income between one to three times the minimum wage (55.9%). Regarding education level, categorized as illiterate, 1–4 years, and ≥ 5 years, 65 participants had ≥ 5 years of education (55.1%) (**Table 1**).

**TABLE 1:** Percentage distribution of sociodemographic variables among older Brazilian patients undergoing hemodialysis. Ribeirão Preto, SP, Brazil, 2025 (*n* = 118)

Variables	Categories	n	%
Sex	Male	82	69.5
	Female	36	30.5
Age	60–69 years	60	50.8
	70–79 years	49	41.5
	80+ years	9	7.6
Ethnicity	White	75	63.6
	Non-White	43	36.4
Marital status	With a stable partner	64	54.2
	Without a stable partner	53	44.9
Income	Up to one the minimum wages	31	26.3
	From one to three the minimum wages	66	55.9
	Four or more the minimum wages	19	16.1
Education level	Illiterate	7	5.9
	From 1 to 4 years	46	39
	Equal or more than 5 years	65	55.1

Regarding social support domains, the informational support had the lowest mean score 81.69 (± 24.60), followed by positive social interaction support 82.71 (± 23.08), affective support 85.25 (± 22.62), material support 92.92 (± 14.10), and emotional support 93.44 (± 12.97). Regarding family APGAR scores, 83.9% had good family functioning, 9.3% had moderate family dysfunction, and 6.8% had severe family dysfunction (**Table 2**).

**TABLE 2:** Descriptive analysis of social support and family functioning. Ribeirão Preto, SP, Brazil, 2025, (*n* = 118)

Variables	Min	Max	Med	Mean	Standard Deviation
Social support					
Material support	30	100	100	92.92	± 14.10
Affective support	53	100	100	93.44	± 12.97
Emotional support	20	100	100	85.25	± 22.62
Informational support	20	100	95	81.69	± 24.60
Positive social interaction support	20	100	95	82.71	± 23.08

Analysis of social support scores across family functioning levels revealed significant differences for all social support domains (*P* < 0.001). Participants categorized as having severe family dysfunction reported the lowest levels of social support. These levels were evident, especially regarding positive social interaction support. The level was the lowest in the severe family dysfunction group (mean = 55 [± 28.66]), whereas the mean for the good family functioning group was 87.78 (± 19.04). For informational support, the severe family dysfunction group had a mean of 57.5 (± 32.4), whereas the good family functioning group had a mean of 86.11 (± 21.56). The domain of emotional support had a mean score for severe dysfunction of 61.25 (± 28.62), whereas the group of good family function had a mean of 91.26 (± 16.10). Similar trends were observed across the other domains; material support had a mean of 80 (± 22.83) for the severe dysfunction group and 95.15(± 11.54) for the good functioning group, followed by affective support, with a mean of 83.37 (± 18.11) for the severe dysfunction and a mean of 96.22 (± 10.05) for the good functioning group. These findings collectively underscored that dysfunctional families were strongly associated with a diminished perception of social support (**Table 3**).

**TABLE 3:** Distribution statistics of the mean scores of social support according to the MOS questionnaire and levels of Family Functioning (APGAR). Ribeirão Preto, SP, Brazil, 2025 (*n* = 118)

Questionnaire	Categories	n	Mean	SD	P value
Material support	Severe family dysfunction	8	80	22.83	< 0.001a,b
	Moderate family dysfunction	11	82.27	18.62	
	Good family function	99	95.15	11.54	
Affective support	Severe family dysfunction	8	83.37	18.11	< 0.001a,b
	Moderate family dysfunction	11	75.73	15.63	
	Good family function	99	96.22	10.05	
Emotional support	Severe family dysfunction	8	61.25	28.62	< 0.001a,b
	Moderate family dysfunction	11	48.64	24.09	
	Good family function	99	91.26	16.10	
Positive social interaction Support	Severe family dysfunction	8	55	28.66	< 0.001a,b
	Moderate family dysfunction	11	57.27	22.84	
	Good family function	99	87.78	19.04	
Informational support	Severe family dysfunction	8	57.5	32.40	< 0.001a,b
	Moderate family dysfunction	11	59.54	24.23	
	Good family function	99	86.11	21.56	

^a^Severe family dysfunction ≠ Good family function; bModerate family dysfunction ≠ Good family function. SD = standard deviation.

In the correlational analysis, a moderate positive correlation was confirmed between family functioning and each domain of social support, with a statistically significant threshold of *P* < 0.001 for all domains of social support (**Table 4**).

**TABLE 4:** Spearman’s correlation coefficients between Family Functioning (APGAR) and Social Support (MOS) scores, according to its dimensions (MS, AS, ES, PSIS, IS). Ribeirão Preto, SP, Brazil, 2025 (*n* = 118)

		MS	AS	ES	PSIS	IS
APGAR	r	0.327	0.425	0.518	0.466	0.421
	P value	< 0.001	< 0.001	< 0.001	< 0.001	< 0.001

MS = material support; AS = affective support; ES = emotional support; PSIS = positive social interaction support; IS = informational support.

## DISCUSSION

Consistent with the study’s objectives, the results demonstrated that most hemodialysis participants were men (69.5%), aged 60–69 years (50.8%), of white ethnicity (63.6%), and had a stable partner (54.2%). These findings are consistent with those of both Brazilian and international studies showing a predominance of men and older adults in hemodialysis programs, reflecting the epidemiological profile of CKD in adult and older aged populations. A substantial proportion of participants reported low income (55.9%), earning between one and three times the minimum wages, in line with the Instituto Brasileiro de Geografia e Estatística (IBGE)^18^ classifications of economically vulnerable populations, reinforcing the association between CKD and unfavorable socioeconomic conditions. Notably, 55.1% of participants had completed ≥ 5 years of schooling, corroborating international findings that suggest a relatively high educational level attainment among hemodialysis patients.^9,17,19^

Overall mean scores for social support were high, indicating generally positive perceptions. However, the mean scores for informational support (81.69 ± 24.60) and positive social interaction (82.71 ± 23.08) were lower than those for emotional (93.44 ± 12.97) and tangible support (92.92 ± 14.10). This pattern aligns with prior reports and may reflect the constraints of prolonged treatment, restricted social networks, and reliance on specialized information for disease management.^4,9^

Family functioning, measured by the Family APGAR scale, revealed that 83.9% of participants had functional families, whereas 9.3% and 6.8% reported moderate and severe dysfunction, respectively. Consistent with the findings of Safi et al.,^5^ higher family functioning was associated with greater perceived social support, highlighting the critical role of the family as a source of emotional and instrumental support during hemodialysis. Analysis by family functioning level revealed statistically significant differences across all support domains (P < 0.001). Participants from families with severe dysfunction had the lowest scores in terms of positive social interaction (55 ± 28.66) and informational support (57.5 ± 32.4), whereas those from functional families scored substantially higher (87.78 ± 19.04 and 86.11 ± 21.56, respectively). These findings suggest that the family environment exerts a direct influence on social support perception, reinforcing its mediating role in psychological and social well-being.

Correlation analyses demonstrated moderate positive associations between family functioning and all social support domains (P < 0.001), indicating that better family dynamics are linked to higher perceived social support. This is consistent with the findings of Wang et al.,^10^ who reported a longitudinal relationship between family resilience and social support in patients undergoing kidney treatment, emphasizing the interplay between familial and psychosocial factors.

Recent studies have further underscored the potential of targeted interventions to enhance family support and resilience. Moradi et al.^20^ showed that family-centered virtual programs improved mental health outcomes in patients and caregivers, demonstrating that remote interventions could effectively strengthen family support. Similarly, Supriati et al.^21^ found a significant correlation between peer social support and family psychological resilience, suggesting that interactions with individuals facing similar challenges reinforce coping capacity. Wang et al.^10^ highlighted the mediating roles of family functioning and self-efficacy in the relationship between depression and resilience, while Anita et al.^22^ demonstrated that perceived family support was associated with lower depressive symptoms and improved health-related quality of life.

These findings provide valuable insights but should be interpreted in light of several limitations. The cross-sectional design precludes causal inferences, and reliance on self-reported measures may have introduced response biases. Additionally, the context-specific sample also limits generalizability to other populations or cultural settings. Future research should employ longitudinal designs, explore the efficacy of interventions across diverse sociodemographic contexts, and evaluate their impact on both psychosocial and clinical outcomes.

Overall, the results indicate that adequate family functioning is strongly related to positive perceptions of social support, particularly emotional and tangible support, among patients undergoing hemodialysis. These findings highlight the importance of multiprofessional care strategies that strengthen support networks, actively involve families, and develop coping mechanisms to promote resilience and overall well-being in individuals with CKD.

## CONCLUSION

The findings of this study indicate a moderate correlation between social support and family functioning in older adults undergoing hemodialysis. The limited depth of existing literature on family functionality and social support in this population constrains the corroboration of our findings, underscoring the importance of future research focused on these variables to better inform clinical practice and theoretical development.

## Data Availability

All data generated or analyzed during this study are included in this published article

## References

[1] World Health Organization Noncommunicable diseases.

[2] Brazil. Boletim Epidemiológico (2024). Cenário da doença renal crônica no Brasil no período de 2010 a 2023.

[3] Kidney Disease: Improving Global Outcomes (KDIGO) CKD Work Group (2024). KDIGO 2024 Clinical Practice Guideline for the Evaluation and Management of Chronic Kidney Disease. Kidney Int.

[4] Sułkowski L, Matyja A, Matyja M (2024). Social support and quality of life in hemodialysis patients: a comparative study with healthy controls. Medicina.

[5] Safi F, Namdar Areshtanab H, Ghafourifard M, Ebrahimi H (2024). The association between self-efficacy, perceived social support, and family resilience in patients undergoing hemodialysis: a cross-sectional study. BMC Nephrol.

[6] Kisomi ZS, Taherkhani O, Mollaei M (2024). The moderating role of social support in the relationship between death anxiety and resilience among dialysis patients. BMC Nephrol.

[7] Hulu T, Budi NP, Sari RP (2021). Relationship of family support with quality of life of hemodialyzed patients using study literature review method. J Vocat Nurs.

[8] Pratiwi WN, Gayatri PR, Astutik WS, Pratama YG, Ayyaroh FA (2023). The role of family support in stress and anxiety conditions in the elderly with chronic kidney disease underwent hemodialysis therapy. Adi Husada Nurs J.

[9] Santos DGM, Pallone JM, Manzini CSS, Zazzetta MS, Orlandi FS (2021). Relationship between frailty, social support and family functionality of hemodialysis patients: a cross-sectional study. Sao Paulo Med J.

[10] Wang Y, Qiu Y, Ren L (2024). Social support, family resilience and psychological resilience among maintenance hemodialysis patients: a longitudinal study. BMC Psychiatry.

[11] Ajzen I, Fishbein M (1980). Understanding attitudes and predicting social behavior.

[12] Sherbourne CD, Stewart AL (1991). The MOS social support survey. Soc Sci Med.

[13] Andrade CR, Chor D, Faerstein E (2005). Social support and breast self-examination in the Pró-Saúde Study. Rev Saude Publica.

[14] Smilkstein G (1978). The family APGAR: a proposal for a family function test and its use by physicians. J Fam Pract.

[15] Duarte YAO (2001). Família: rede de suporte ou fator estressor – *a* ótica de idosos e cuidadores familiares [doctoral thesis]. São Paulo: Escola de Enfermagem, Universidade de São Paulo;.

[16] Levin J, Fox JA (2004). Elementary statistics in social research. 9th ed. São Paulo: Pearson Prentice Hall;.

[17] Paula EA, Roth JM, Schwartz E, Lima Spagnolo LM, Lise F (2022). Perfil sociodemográfico e clínico de usuários em hemodiálise no sul do Rio Grande do Sul, Brasil. Enferm Actual Costa Rica.

[18] Instituto Brasileiro de Geografia e Estatística (IBGE) Demographic Census 2022: population and households: preliminary results.

[19] Alatawi AA, Alaamri M, Almutary H (2024). Social support and adherence to treatment regimens among patients undergoing hemodialysis. Healthcare.

[20] Moradi M, Amiri M, Daneshi S (2024). Investigating the effectiveness of a virtual family-centered support intervention on the mental health of hemodialysis patients and their family caregivers during the COVID-19 pandemic. Open Public Health J.

[21] Supriati L, Sunarto M, Ulya I (2024). The role of peer social support on family psychological resilience in caring for chronic kidney disease patients receiving hemodialysis. Healthcare Low-Resour S.

[22] Anita DC, Alfrizki ES, Suprayitno E, Fadlilah S, Wantonoro W (2025). Family support, depression, and health-related quality of life of hemodialysis patients. Turk J Nephrol.

